# A High Dose Intravenous Immunoglobulin Therapy for Women with Four or More Recurrent Spontaneous Abortions

**DOI:** 10.5402/2012/512732

**Published:** 2012-09-11

**Authors:** Hideto Yamada, Masamitsu Takeda, Yoko Maezawa, Yasuhiko Ebina, Ryoichi Hazama, Kenji Tanimura, Yukio Wakui, Shigeki Shimada

**Affiliations:** ^1^Department of Obstetrics and Gynecology, Kobe University Graduate School of Medicine, Kobe 650-0017, Japan; ^2^Department of Obstetrics, Hokkaido University Graduate School of Medicine, Sapporo 060-8638, Japan; ^3^KKR Sapporo Medical Center, Sapporo, Japan; ^4^Mommy's Clinic Chitose, Chitose, Japan

## Abstract

Recurrent spontaneous abortion (RSA) may have immunological etiology. The aim of this study was to assess the efficacy of a high dose intravenous immunoglobulin (HIVIg) therapy, in which 20 g of intact type immunoglobulin was infused daily for 5 days during early gestation, for women who had a history of four or more consecutive spontaneous abortions of unexplained etiology. A total of 60 pregnant RSA women underwent HIVIg therapy, and the pregnancy outcome was assessed. The live birth rate was 73.3% (44/60). Fifteen pregnancies ended in spontaneous abortion, and one ended in intrauterine fetal death. In 11 of the 15 spontaneous abortions, fetuses had abnormal chromosome karyotype. When the 11 pregnancies with abnormal chromosome karyotype were excluded, the live birth rate was as high as 89.8% (44/49). The HIVIg therapy may be effective for severe cases of unexplained RSA.

## 1. Introduction

Recurrent spontaneous abortion (RSA) is defined as the loss of three or more consecutive pregnancies in the first trimester [1]. The RSA affects 1–1.8% of women [1, 2]. A wide variety of causes participate in the pathogenesis of RSA, including uterine anomalies, cervical incompetence, autoimmune diseases, antiphospholipid antibody, chromosomal abnormalities of couples, thrombophilic disorders, endocrinological abnormalities, and microbial infections [2, 3]. For example, parental chromosome abnormalities represented by balanced type translocations are associated with approximately 4% of couples with RSA compared with 0.2% in normal population [4]. However, the etiology in approximately 50% of RSA is unknown, therefore designated as unexplained RSA. It is postulated that they have immunological etiology [1]. 

The precise mechanism underlying the pathology of RSA remains poorly understood. In this context, no standard therapeutic modality for unexplained RSA have been established so far, despite several lines of evidence indicating some therapeutic efficacy of unfractionated heparin or low molecular weight heparin with or without low dose aspirin, paternal lymphocyte immunization, intravenous immunoglobulin (IVIg), predonisolone, and progestin [5–7]. In the recently emerging literature, novel clinical approaches with the use of tumor necrosis factor inhibitors [8, 9] and granulocyte colony-stimulating factor [10] have been conducted for the treatment of RSA. 

We for the first time developed a high dose intravenous immunoglobulin therapy (HIVIg) during early gestation for severe cases with RSA of unexplained etiology in 1993 and previously reported the efficacy in a preliminary study [11]. 

## 2. Materials and Methods

### 2.1. Patients

This prospective study was performed as a multicenter study in Japan, and conducted with informed consent from all of the subjects. The study was approved by the institutional ethical boards of the Kobe University Hospital. During the period between 1993 and 2010, RSA women were admitted to the study if they met all of the following requirements. Subjects must have (i) a history of four or more consecutive spontaneous abortions in the first trimester, (ii) unexplained etiology of RSA and (iii) no allergy for immunoglobulin or IgA deficiency disease. 

All patients underwent examinations of ultrasound, hysterosalpingography, endometrial biopsy, and conventional blood analyses for RSA screening and were diagnosed as having RSA of unexplained etiology. The blood analyses included chromosome karyotypes of couple; measurements of progesterone in mid-luteal phase, prolactin, thyroid, liver, kidney functions, hemostatic coagulation factors such as d-dimer, factor XII, protein C, protein S; and autoimmune factors such as antinuclear antibody, complements, anticardiolipin, *β*2-glycoprotein I-dependent anticardiolipin antibodies, and lupus anticoagulant. 

### 2.2. Immunoglobulin Therapy

They underwent HIVIg therapy (intact type immunoglobulin 20 g daily in the course of 5 days; a total dosage of 100 g) with a written informed consent immediately after a gestational sac was detected in the uterus by ultrasound. Medications of intact type immunoglobulin including Venoglobulin-IH (Benesis, Osaka), Kenketsu glovenin-I (Nihon Pharmaceutical Co., Tokyo), and Sanglopor (CSL Behring, Tokyo) were used.

### 2.3. Pregnancy Outcome

When the index pregnancy ended in spontaneous abortion again, the patient was suggested undergoing chromosome analysis of the villi. Information about complications and pregnancy outcome was collected from medical records. Mann-Whitney *U* tests were used for the comparison between live birth and spontaneous abortion groups. 

## 3. Results

We had conducted HIVIg therapy in 60 RSA women with the ages ranging from 23 to 44 years old who had a history of 4 to 8 spontaneous abortions, and confirmed pregnancy outcome (Table 1). The live birth rate was 73.3% (44/60). One pregnancy ended in intrauterine fetal death at 31 weeks of gestation due to sudden abruptio placenta and severe pregnancy induced hypertension that developed within several hours in one day. This case had uneventful clinical course without abnormal laboratory findings. Fifteen pregnancies ended in spontaneous abortions consisting of 2 spontaneous abortions of a fetus with normal chromosome karyotype, 11 spontaneous abortions of a fetus with abnormal chromosome karyotype (SAAK), and 2 with unknown karyotype. It was impossible to assess the efficacy of HIVIg among the 11 SAAK fetuses who were destined to die. If the 11 pregnancies resulting in SAAK were excluded, the live birth rate was as high as 89.8% (44/49).

Pregnancy complications including premature delivery (18.2%) and intrauterine fetal growth restriction (13.6%) were observed. Fetal anomaly of cleft lip was seen in one case. The adverse effects of HIVIg in the mothers including rash/fever (6.7%) and the elevation of d-dimer levels (6.7%) were found (Table 1).

Table 2 shows patient characteristics (*n* = 60) and the comparison between live birth (*n* = 44) and spontaneous abortion (*n* = 15) groups excluding one intrauterine fetal death. The number of previous abortions, percentages of primary RSA, or gestational week of HIVIg therapy was not statistically different between the live birth and spontaneous abortion groups. Maternal age of spontaneous abortion group was relatively higher than that of live birth group, but without statistical significance (*P* = 0.067). In a total 60 patients, 4 cases with concurrent IVF-ET therapy and 9 cases with clomiphene or hMG-hCG treatment were included. 

## 4. Discussion

It is well acknowledged that intravenous use of immunoglobulin is practically effective, and this therapy has long been applied to a wide variety of immune-mediated diseases such as idiopathic thrombocytopenic purpura, Guillain-Barré syndrome, Kawasaki's disease, and myasthenia gravis [12, 13]. Several mutually nonexclusive mechanisms of action, which include the suppression of inflammation and modification of Fc receptor, T cell, B cell, or macrophage functions, are proposed to account for the immunoregulatory effects of the IVIg therapy [13]. 

To assess the efficacy of IVIg in women with unexplained RSA, randomized, double-blind, and placebo-controlled trials with use of a medium dose of IVIg therapy, in which 20–40 g of Ig/person is infused weekly or every 2–4 weeks during early and midgestation, have been performed in 1990s [14–19]. Conclusions drawn from these IVIg trials are controversial. Thereafter, reports of meta-analysis [20] and systematic review [21] concerning efficacy of IVIg therapy suggested that a medium dose of IVIg was effective among women with secondary RSA. On the other hand, our group for the first time tried HIVIg therapy for severe cases of unexplained RSA, in which 100 g of intact type immunoglobulin was infused intravenously over the course of 5 days at approximately 5 weeks of gestation. We expected immunomodulatory effects of a high dose immunoglobulin. We previously reported high live birth rates among small-scale subjects in the preliminary HIVIg studies [11, 22]. Thereafter, the study was continued and consequently we confirmed the high birth rate and the efficacy of HIVIg for severe cases of unexplained RSA in the present study. When 11 pregnancies resulting in spontaneous abortion with abnormal chromosome karyotype were excluded, the live birth rate was as high as 89.8%. The percentage of spontaneous abortion with abnormal chromosome karyotype was 18.3% (11/60). Maternal age of spontaneous abortion group might be related to relatively high incidence of chromosome abnormality. HIVIg therapy was well tolerated in most of these subjects, and none of them discontinued HIVIg therapy due to serious adverse effects. A successful HIVIg therapy (immunoglobulin 400 mg/kg daily in the course of 5 days; a total approximately 100 g) on a pregnant woman with antiphospholipid syndrome who had lupus anticoagulant and a history of 9 RSA was first reported in 1988 [23]. However, there has been no report concerning HIVIg therapy on unexplained RSA except for our literatures [11, 22].

Several immunological mechanisms underlying the pathophysiology of RSA and action of IVIg have been suggested. IVIg modulate NK cell dynamics and cytokine production. The immunomodulatory effects of IVIg on NK cells in RSA women include the reduction of peripheral NK cell number and cytotoxicity [22, 24–27] and the increase in expression of inhibitory receptor CD94 on NK cells [28]. IVIg therapy in RSA women was shown to drive a Th1/Th2 balance toward Th2 shift [29, 30] and to increase serum cytokine [30] and G-CSF levels [31]. 

Using a mouse model of immunological reproductive failure, we for the first time demonstrated that a high dose of intact type-immunoglobulin but not Fab-immunoglobulin restored the fecundity and that spleen cells adoptively transferred from immunoglobulin injected nonpregnant donors to pregnant recipient mice of reproductive failure completely restored the fecundity. The restoration of the fecundity was concomitant with reduction of TNF-*α* and IFN-*γ* expressions in the placentas. CD11b+ macrophages transferred from donor mice accumulated selectively in the placentas of recipient mice, suggesting that macrophages as mediator and/or effector cells may play a key role in mechanisms of HIVIg efficacy [32]. These observations may explain a favorable pregnancy outcome in HIVIg treated RSA women who have immunological abnormalities. These data with use of HIVIg strongly support the evidence showing the usefulness of this therapy as immune modifier, when performed during early gestation. It is likely that HIVIg correct etiological immunological abnormalities in RSA women. 

The results of the present study included several uncertainties. Enrollment of subjects in the study design was not randomized or placebo-controlled. Patients did not undergo measurements of relatively new antiphospholipid antibodies including antiphosphatidylethanolamine and phosphatidylserine-dependent antiprothrombin antibodies. During long study period, a variety of the lot of immunoglobulin made by different manufacturers was used for the therapy. HIVIg therapy has infection risks of small viruses such as human parvovirus B19. The cost of HIVIg is much higher than that of aspirin-heparin therapy. A randomized placebo-controlled study should be further performed to confirm the efficacy of HIVIg (20 g daily in 5 days; a total 100 g) on severe cases of RSA with unexplained etiology or intractable RSA with resistance to aspirin-heparin therapy.

## Figures and Tables

**Table 1:** Pregnancy outcome, complications and adverse effects in 60 women with severe cases of unexplained recurrent spontaneous abortion who underwent a high dose intravenous immunoglobulin therapy.

Pregnancy outcome	Number	Complications	Number (%)
Live birth	44	Premature delivery	8/44 (18.2)
IUFD at 31st week	1	IUGR	6/44 (13.6)
Spontaneous abortion	15	Anomaly (cleft lip)	1
Normal chromosome karyotype	2	Adverse effects on mothers	
Abnormal chromosome karyotype	11	Rash and fever	4/60 (6.7)
Unknown karyotype	2	Elevation of d-dimer	4/60 (6.7)

IUFD: intrauterine fetal death; IUGR: intrauterine growth restriction.

**Table 2:** Patient characteristics and the comparison between live birth and spontaneous abortion groups.

Age (year-old)	Number of previous abortions	Percentage of primary RSA	Gestational week of immunoglobulin injection	Gestational week of delivery or abortion	Birth weight (g)
Total *n* = 60					
35 (23–44)	4.5 (4–8)	85.0%	5 (4–7)		

Outcome					
Live birth *n* = 44					
34.5 (23–42)	4 (4–8)	84.1%	5 (4–7)	38 (29–41)	2815 (636–3570)

Spontaneous abortion *n* = 15					
37 (27–44)*	5 (4–8)	86.7%	5 (4–6)	8 (6–9)	—

Median (range).

*P = 0.067 compared with age of live birth group.

RSA: recurrent spontaneous abortion.
